# Differential Effects of Anxiety on Internet Gaming Disorder: A Large-Scale Cross-Sectional Survey

**DOI:** 10.3389/fpsyt.2021.802513

**Published:** 2022-01-28

**Authors:** Xia Huang, Hong-xia Shi, Hui-qin Li, Wan-jun Guo, Dan Luo, Jia-jun Xu

**Affiliations:** ^1^West China School of Nursing, West China Hospital, Sichuan University, Chengdu, China; ^2^Mental Health Center, West China Hospital, Sichuan University, Chengdu, China; ^3^Department of Pharmacy, West China Hospital, Sichuan University, Chengdu, China

**Keywords:** cross-sectional survey, internet gaming disorder, anxiety, addictive behavior, IgD

## Abstract

**Background:**

Internet gaming disorder (IGD) has become a serious public health problem in East Asia, and studies have reported IGD to be significantly associated with anxiety, but no causal relationship between the two has yet been demonstrated. Children are at high risk of developing IGD, however, previous studies have principally focused on the condition in adults and adolescents and reported non-clinical samples. A large-scale survey is needed to research and evaluate IGD and anxiety in children and adolescents to understand the current situation of IGD in children and explore the impact of IGD on anxiety.

**Methods:**

A cross-sectional study using an online questionnaire was conducted between March 1 and July 31, 2021. A total of 10,479 school children and adolescents in the western provinces of China were selected by convenience sampling. A questionnaire was used to collect data anonymously. The questionnaire covered IGD and the Revised Children's Manifest Anxiety Scale (RCMAS). Welch's ANOVA Test and Games-Howell test were used to test for differences in anxiety levels between IGD groups. Poisson regression analysis was used to further investigate the key predictors of IGD.

**Results:**

3.2% of participants (*n* = 334) (95% CI: 2.9–3.2%) were classified as at high risk of presenting with IGD, 71.1% (*n* = 7,454) (95% CI: 70.3–72.0%) were classified as low-risk players, and 25.7% (*n* = 2,691) (95% CI: 24.9–26.5%) were classified as non-gaming. The average RCMAS score was (7.18 ± 7.534). The high-risk group had a higher total score RCMAS, as well as scoring higher in its three dimensions. Regression analysis using gender, age, and total RCMAS score as independent variables, and risk of IGD as a dependent variable showed that the odds ratio (OR) for gender was 2.864 (95% CI: 2.267–3.618), and the OR for total RCMAS score was 1.101 (95% CI: 1.087–1.114). The OR for age was not statistically significant.

**Conclusion:**

Anxiety was a predictor of IGD, with statistically significant group differences in total anxiety, as well as the dimensions of physiological anxiety, social correlation, and sensitivity. The timely assessment of anxiety in children and adolescents, training social skills, and facilitating effective integration into society could be effective ways of reducing the incidence and impact of IGD.

## Introduction

With the rapid development of the Internet, the internet gaming market has also grown rapidly, bringing China US$12 billion in revenue in 2013 alone (1). This has also attracted the attention of mental health practitioners to the growing problem of Internet addiction (1–4). However, Internet addiction is a general term covering various undesirable consequences of problematic Internet overuse (5). Internet Gaming Disorder (IGD) is the most common type of Internet addiction (6), and is associated with impulsivity, attention deficit disorder (7), and can lead to loneliness and reduced life satisfaction and academic performance (8). IGD has become a serious public health problem in East Asia (9). As of December 2018, the incidence of gaming addiction in China reached 27.5% of the total population, representing 30.5% of teenagers (10). Children also commonly experience IGD, with studies reporting the prevalence of IGD to be as high as 38.0% in children (11). IGD is defined as continuous and repeated involvement with video games, often leading to significant disruption to daily life, work, and/or education (Fifth Edition, Diagnostic and Statistical Manual of Mental Disorders, DSM-5). The America Psychiatric Association (APA) proposed adding IGD to the “emerging measures and models” group of mental conditions, acknowledging that further research and evaluation of the condition is highly necessary (12).

Previous studies reporting the evaluation of IGD have used self-reported continuous scales to determine the level of addiction. Few studies report the use of cut-off values or diagnostic criteria to determine the true clinical significance of IGD. Any IGD score that does not reach clinical diagnostic significance may not be representative of the detrimental impact of clinical symptoms. Further, the number of online gamers is huge, and many have a normal level of social function. This may in part be due to the lack of a clinical diagnosis for IGD.

Anxiety is one of the most common mental health disorders treated in primary care (13), with an annual incidence of 1–4% and a lifetime prevalence of 4–7% (14, 15). Symptoms of anxiety have been found to be significantly higher in individuals with IGD (16), and research has revealed a significant correlation between IGD and anxiety (17). Another study found IGD and anxiety to be comorbid (18). When research findings are divided into anxiety subtypes, more attention has been given to the relationship between social anxiety and IGD. Players with symptoms of social anxiety are thought to create and maintain the safety and control of their environment through the game to compensate for their lack of social interaction in real life (19, 20). The interaction of other forms of anxiety with IGD is less studied. IGD has been suggested to be a moderating factor, with gaming sometimes being used to alleviate the symptoms of anxiety and depression by providing escapism (21, 22). Previous studies have found people with anxiety disorders to be more likely to develop addictive behaviors through the need for coping mechanisms such as alcohol abuse (23–25). The question of whether anxiety symptoms increase the risk of IGD requires more research.

Children are at high risk of IGD. Previous studies have mostly focused on adults and adolescents and studied non-clinical populations. Few studies have investigated IGD in both children and adolescents simultaneously (26–28), and sample sizes have been modest. To sample a sufficient number of positive IGD patients through screening the general population requires large-scale investigation (17). As such, a large-scale survey and evaluation of the prevalence of IGD in children and adolescents are needed to explore the impact of anxiety on IGD. The research hypotheses are that anxiety and IGD are significantly related, and the higher the level of anxiety, the higher the risk of IGD. Therefore, the large-scale cross-sectional survey was used to verify the hypotheses, and the specific objectives are as follows: (1) to understand the current prevalence of IGD in children; and (2) to explore the impact of anxiety on IGD, providing references for the prevention and treatment of anxiety and IGD in children and adolescents.

## Materials and Methods

### Participants

The study inclusion criteria were: (1) children aged between 9 and 18 years; (2) children whose parents and themselves agreed to participate in the study; and (3) children who were able to complete the questionnaire independently. The study exclusion criteria were: (1) children with a medical diagnosis of a learning disability, developmental delay, or dyslexia; or (2) children who did not want to participate in the study. The samples for this study are from 12 primary and middle schools. The researchers contacted the principal of each school, and after obtaining the principal's approval, we went to the school to recruit samples.

### Measures

#### Sociodemographic Data

Demographic data collected included the participant's gender, age, family structure, whether they were an only child, the education level of their parents, and their household monthly income.

#### Nine-Item Internet Gaming Disorder Scale Short Form

The nine-item Internet Gaming Disorder Scale Short Form (IGDS-SF9), developed by Pontes and Griffiths, is a short self-report scale that is used to measure the severity of IGD based on the Diagnostic and Statistical Manual of Mental Disorders (DSM-5) criteria (29, 30). Confirmatory factor analysis (CFA) of the IGDS-SF9 demonstrated that it has a unidimensional structure with satisfactory psychometric properties including internal consistency (Cronbach's α of 0.9), and criterion and construct validity among university students in Hong Kong (31) and primary school students in mainland China (32). The IGDS9-SF contains nine questions that are answered using a 5-point Likert scale: 1 = never; 2 = rarely; 3 = sometimes; 4 = often; and 5 = very often. A score is obtained by summing across all responses and can range from 9 to 45, with a higher score indicating a higher degree of gaming disorder (33). In the present study, if a participant answered “No” to the question “Have you played computer games in the past 12 months, regardless of the device (computer, tablet, mobile phone) used?,” they were considered to non-gamer. In accordance with the APA's recommendations, participants who answered either “frequently” or “very often” to at least five of the nine items in the questionnaire were operationally defined as being likely to receive a positive IGD diagnosis, and formed the “high-risk group.” The remaining participants were defined as the “low-risk group” (30).

#### The Revised Child Manifest Anxiety Scale

The Revised Child Manifest Anxiety Scale (RCMAS), published by Reynolds and Richmond (34), was based on the Child Manifest Anxiety Scale (CMAS) designed by Casteneda, McCandless, and Palermo (35). The RCMAS is used to assess anxiety in children and adolescents aged 6–19. RCMAS is a self-report instrument where a response of “Yes” indicates that the item describes the subject's feelings or actions, while a response of “No” indicates that the symptom is not present. RCMAS contains 37 items in 4 subscales (factors); physiological anxiety (10 items), worry and oversensitivity (11 items), social concern and concentration (7 items), and lie (9 items). The total anxiety score is computed based on the first three factors (28 items), with a score of 19 or greater suggesting a clinically significant degree of anxiety. The remaining 9 items from the lie scale are designed to measure the degree to which the child is “faking good.”

### Data Collection

The study was conducted over a large geographical area, between March and May, 2021. A total of 12 primary and middle schools from the region participated in the survey. Before collecting data, study information was sent to students, asking them to participate after obtaining written informed consent from their parents or themselves. The survey was predominantly conducted online survey, with some paper copies of the questionnaire provided to those who requested this format.

### Ethical Considerations

All procedures were conducted in accordance with the 1964 Declaration of Helsinki and its later amendments. The study was approved by the Biomedical Ethics Committee of West China Hospital of Sichuan University (Ethics Number: 2019907). All participants were informed about the study and written informed consent was provided by the parents or legal guardians. In addition, participants and their parents were informed that participation was anonymous, all data was to be used only for scientific research, and they had the right to withdraw from the study at any time.

### Statistical Analyses

Incomplete questionnaires were excluded from the analysis. The results of the electronic questionnaire were directly exported. Data from the questionnaires completed on paper were entered into Epidata 3.1 using a two-person entry method. These two repositories were then merged into a single spreadsheet in SPSS 24.0 for statistical analysis. Frequencies were used to report participants' sociodemographic characteristics and IGD scores. RCAMS scores were reported using the mean, standard deviation, and interquartile range. For unordered categories data, such as gender, family structure and whether one-child or not, Mann—Whitney U was used to compare the differences in rates between groups, while for ordinal categorical data, such as age, family income, and parents' education level, the Spearman correlation test was used to compare the differences in game addiction among the subjects under different demographic characteristics. The average scores of anxiety levels between different IGD groups are tested for homogeneity of variance. If the variances are uniform, analysis of variance is used to compare the differences in anxiety levels between different groups. If the variances are not uniform, Welch's ANOVA Test is used to compare different gaming addiction groups. If the difference in anxiety levels between the groups is significant, the Games-Howell test (36) will be used to further compare the two groups. On the basis of demographic differences and anxiety level differences, statistically significant factors were used as dependent variables for regression analysis. If the model fitting test and parallelism test are passed, the ordered categorical variable logistic regression is adopted. If the model fails, according to the actual clinical significance of the variable, non game players and low-risk game players are combined and defined as non high-risk game player group, which is assigned as 0, and the high-risk game player group is assigned as 1, and then Poisson region analysis is used to further explore the key predictors of IGD.

## Results

### Descriptive Statistics

A total of 10,501 participants completed part of the survey. After excluding invalid questionnaires, the remaining 10,479 respondents were included in the analysis. Invalid questionnaires were defined as those having the same responses for more than 90% of the answers except for basic demographic data. The age range of participants was 9 to 18 years, most of whom were in the 13–15 years group (54.19%), with uniform gender distribution (50.55% of participants were male), and most of them were the only child in the household (60.21%). See Table 1 for details.

**Table 1:** Participant sociodemographic characteristics.

**Sociodemographic characteristics**	***N* (%)**
**Gender**	
Male	5,297 (50.55)
Female	5,182 (49.45)
**Age (years)**	
9–12	3,852 (36.76)
13–15	5,679 (54.19)
16–18	948 (9.05)
**Family structure**	
Single-parent families and others	1,537 (14.67)
Two-parent family	8,942 (85.33)
**Only child**	
Yes	4,170 (39.79)
No	6,309 (60.21)
**Education level of father**	
Junior school or below	3,679 (35.11)
Senior schools	2,029 (19.36)
Vocational schools	2,784 (26.57)
Bachelor or above	1,987 (18.96)
**Education level of mother**	
Junior school or below	3,926 (37.47)
Senior schools	1,846 (17.62)
Vocational schools	2,656 (25.35)
Bachelor or above	2,051 (19.57)
**Monthly family income**	
8,000 above	2,972 (28.36)
6,000–8,000	2,671 (25.49)
4,000–6,000	2,736 (26.11)
2,000–4,000	1,778 (16.97)
2,000 below	322 (3.07)

Harman's single factor test was used to test for common method bias (28). The proportion of the total variance explained was 27.121%. As this was less than the threshold value of 40%, it was considered that there was no significant common method bias.

### The Prevalence of IGD and the Influence of Sociodemographic Characteristics on IGD

The prevalence of IGD was estimated based on the IGDS9-SF results. 3.2% of participants (*n* = 334) (95% CI: 2.9–3.2%) were classified as high risk, 71.1% (*n* = 7,454) (95% CI: 70.3–72.0%) were classified as low risk, and 25.7% (*n* = 2,691) (95% CI: 24.9–26.5%) were classified as non-gamers.

The Mann–Whitney *U*-test was used to investigate whether IGD group was associated with gender, family structure, and being an only child. The Goodman-Kruskal Gamma test was used to investigate whether IGD group was associated with age, parental education level, and family income. There were no significant associations between IGD group and family structure, parental education level, or family incomes. There were associations between IGD group and both gender and age, with a higher proportion of the high-risk group being male and older (see Table 2).

**Table 2:** Estimated IGD prevalence in different participant demographic groups.

**Characteristics**	**Non-gamers *N* (%)**	**Low risk gamers *N* (%)**	**High risk gamers *N* (%)**	***Z/*G**	***P*-value**
**Gender**					
Male	1,041 (19.7)	4,028 (76.0)	228 (4.3)	−15.136	0.001
Female	1,650 (31.8)	3,426 (66.1)	106 (2.0)		
**Age, year**					
9–12	1,062 (27.6)	2,693 (69.9)	97 (2.5)	0.068	0.000
13–15	1,390 (24.5)	4,084 (71.9)	205 (3.6)		
16–18	239 (25.2)	677 (71.4)	32 (3.4)		
**Family structure**					
Single-parent families and others	397 (25.8)	1,080 (70.3)	60 (3.9)	−0.361	0.718
Two-parent family	2,294 (25.7)	6,374 (71.3)	274 (3.1)		
**Only child**					
Yes	1,057 (25.3)	2,970 (71.2)	143 (3.4)	−0.916	0.360
No	1,634 (25.9)	4,484 (71.1)	191 (3.0)		
**Education level of father**					
Junior school or below	908 (24.7)	2,652 (72.1)	119 (3.2)	−0.029	0.068
Senior schools	529 (26.1)	1,438 (70.9)	62 (3.1)		
Vocational schools	708 (25.4)	1,993 (71.6)	83 (3.0)		
Bachelor or above	546 (27.5)	1,371 (69.0)	70 (3.5)		
**Education level of mother**					
Junior school or below	978 (24.9)	2,845 (72.5)	103 (2.6)	−0.024	0.128
Senior schools	477 (25.8)	1,310 (71.0)	59 (3.2)		
Vocational schools	648 (24.4)	1,898 (71.5)	110 (4.1)		
Bachelor or above	588 (28.7)	1,401 (68.3)	62 (3.0)		
**Monthly family income**					
8,000 above	785 (26.4)	2,074 (69.8)	113 (3.8)	−0.019	0.211
6,000–8,000	712 (26.7)	1,888 (70.7)	71 (2.7)		
4,000–6,000	674 (24.6)	1,974 (72.1)	88 (3.2)		
2,000–4,000	434 (24.4)	1,289 (72.5)	55 (3.1)		
2,000 below	86 (26.7)	229 (71.1)	7 (2.2)		

### The Association Between Anxiety and IGD

The mean RCMAS score was 7.18 ± 7.534, with a skewed distribution and a median of 4 (P25 = 1, P75 = 12). The worry and oversensitivity subscale was the highest-scoring subscale (see Table 3).

**Table 3:** Anxiety as measured using the RCMAS.

**Anxiety**	**Mean**	**SD**	**Median and interquartile**		
			**25**	**50**	**75**
Total score	7.18	7.534	1.00	4.00	12.00
physiological anxiety	2.17	2.512	0.00	1.00	3.00
worry—oversensitivity	3.38	3.550	0.00	2.00	6.00
social concern and concentration	1.63	2.005	0.00	1.00	3.00

Due to there being unequal variance across groups, Welch's ANOVA was used, and the Games-Howell test was used for *post-hoc* testing, and the results reported in Table 4. The results showed that the high-risk group scored higher than low-risk group and non in the total RCMAS score as well as individual scores in the three dimensions, and the differences are significant (*P* < 0.001).

**Table 4:** Anxiety scores by IGD group.

**Variable**	**Non-gamers (N)**	**Low risk gamers (L)**	**High risk gamers (H)**	***F***	***P***	***Post-hoc***
Total score	4.54 ± 6.493	7.85 ± 7.494	13.51 ± 8.999	327.739	0.001	H>L>N^**^
physiological anxiety	1.39 ± 2.147	2.37 ± 2.511	4.22 ± 3.176	263.598	0.001	H>L>N^**^
worry—oversensitivity	2.20 ± 3.139	3.71 ± 3.554	5.71 ± 3.891	276.752	0.001	H>L>N^**^
social concern and concentration	0.95 ± 1.634	1.78 ± 2.013	3.57 ± 2.479	341.999	0.001	H>L>N^**^

** *p < 0.001*.

### Factors Predictive of IGD

As the dependent variable had three levels (i.e., “non-gamers,” “low-risk” and “high-risk”), an ordered categorical logistic regression was performed, whereby the non-gamers group was assigned a value of 0, the low-risk group assigned a value of 1, and the high-risk group assigned a value of 2. Simultaneous forced entry was used to include all variables that showed group differences in the previous analysis. No collinearity was found between variables (VIF <10). The results of the model fitting were as follows: model fitting information (χ^2^ = 1,285.823, *p* = 0.001) and parallelism test (χ^2^ = 84.725, *p* =0.000), indicating that the model did not pass the parallelism test. Informed by the clinically significant distinction made by the IGD score, the combination of non-gamer and low-risk groups were defined as a “non-high-risk group” and assigned a value of 0, and the high-risk group alone was assigned a value of 1. Poisson regression was used with all dependent variables to investigate group differences in gender, age, and total anxiety score, and the results are reported in Table 5. An omnibus test of model coefficients resulted in a χ^2^ of 293.261 (*P* < 0.001). These results show that gender and total anxiety score were predictive of IGD.

**Table 5:** Differences in risk of IGD among different gender, age and anxiety.

**Variable**	**IGD risk**		**CR (95%)**	**B (*P*-value)**
	**Non-high *N* (%)**	**High *N* (%)**		
**Gender**				
Male	5,069 (95.7)	228 (4.3)	2.846 (2.267–3.618)	1.046 (0.000)
Female	5,076 (98.0)	106 (2.0)	1	
**Age (years)**				
9–12	3,755 (97.5)	97 (2.5)	1	
13–15	5,474 (96.4)	205 (3.6)	1.160 (0.908–1.480)	0.148 (0.234)
16–18	916 (96.6)	32 (3.4)	0.947 (0.634–1.416)	−0.054 (0.791)
Anxiety	6.98 ± 7.388	13.51 ± 8.999	1.101 (1.087–1.114)	0.096 (0.000)

## Discussion

This study investigated the incidence of IGD, and the association between anxiety and IGD, in children and adolescents. These data are informative for the prevention and treatment of IGD in children and adolescents. The prevalence of IGD in 9 to 18-year-olds was 3.2%, in agreement with the results of other studies (27, 28, 37, 38) reporting a prevalence of IGD in adolescents of 1.6–5.2%. However, one study (39) revealed a higher prevalence of IGD in British adolescents of 14.6%. This may have been due to different IGD measurement tools being used in the two studies. The present findings are supportive of the notion that countries and regions that differ in economic status have similar IGD incidence rates. Due to the limited access to computers, tablets, and smartphones in countries with low economic status, this may suggest that the incidence of addictive behaviors may have little association with the attributes of electronic products themselves.

Gender was significantly associated with the prevalence of IGD, and IGD was twice as prevalent in male (4.3%) compared to female (2.0%) respondents. This is consistent with the results of previous work (26) reporting that across multiple age ranges, men spend more time on computer games than women. Other studies (40, 41) support a significant correlation between gender and IGD in children and adolescents. It is a widely-held belief that most games on the market are designed by men for men (2), and game companies target male customers (1), resulting in more male users. Studies have also shown that men typically devote more time to playing games than women do (1, 42), with an average of 13–27.76 h per week (2, 43, 44). This may explain a higher prevalence of IGD in men than women.

This study included both children and adolescents. The association between age and IGD is complex. When participants were divided into non-gamers, low-risk gamers, and high-risk gamers, there was a weak (*G* = 0.068) correlation between age and IGD. However, when participants were divided into non-high-risk and high-risk groups for multiple regression, age did not show any effect on IGD. Previous work (45) has suggested that age has an impact on IGD, with a key demarcation point occurring at 14 years of age due to neurodevelopmental changes (i.e., functional connections in the dorsal striatum) occurring after that age (46). Compared with other age groups, children and adolescents under 14 are more likely to make choices that bring immediate rewards and are less likely to be deterred by future negative consequences (47). This may explain why IGD is significantly associated with age. The findings of the present study show that the effect of age on IGD is unclear, and more studies are needed to confirm the existence of an age cut-off point in the incidence of IGD.

The present findings show no significant association between parental education level and IGD, which is inconsistent with the results of other studies (41). This inconsistency may in part be due to differing levels of socioeconomic status across the countries studied, however, such differences are unlikely to account for all the differences in the findings of the two studies. This may an interesting direction for future scholars to explore. The results also showed that family structure and the number of children in the household had no significant effect on IGD.

Our research shows that anxiety is a predictor of IGD. Total anxiety as measured by the RCMAS, as well as subscale measures of physiological anxiety, social correlation, and sensitivity all significantly differed between IGD groups. The results validate our research hypotheses that anxiety and IGD are significantly related, and the higher the level of anxiety, the higher the risk of IGD. It has been suggested that frequent and excessive gaming is a temporary coping strategy for people experiencing negative mental states (48). Additionally, individuals may use video games to escape negative emotions such as distress, anxiety, and depression (49). Studies have confirmed that anxiety and IGD are co-morbid (50). Physiological anxiety refers to the physical symptoms caused by anxiety, and it is one of the components of anxiety. It has been reported that individuals with IGD experience changes in body perception such as stiffness and uncoordinated body movements (51–54). This may explain why total anxiety score and physiological anxiety were both positively correlated with IGD. It has also been shown that social anxiety is a risk factor for the development and continuation of IGD (55–57). Another study reported a positive two-way relationship between Internet gaming and social anxiety (58). It may be that individuals who are reluctant to communicate with others due to social anxiety participate in games to meet their social needs to a certain extent. Individuals may develop a preference for online interaction, increasing the likelihood of IGD (59, 60). Therefore, timely assessment of anxiety in children and adolescents, together with training social skills, and assisting them in effectively integrating into real life may be effective in reducing the incidence and impact of IGD in children and adolescents.

### Advantages and Limitations

This article reports the findings of a large-scale cross-sectional survey. The vast sample size increases the objectivity and generalizability of the findings. The broad age range of 9–18 years covers childhood and adolescence, thereby providing an understanding of the development of IGD with greater scope for behavioral changes that happen over this age range. The limitations of this study are as following: (1) The results of our study showed that gender and anxiety predicted IGD, but we did not examine any potential sex-specific interactions with types of anxiety, which limits our discussion of our results; (2) A self-report tool was used, which may be affected by social desirability bias causing participants to underestimate their own gaming addiction. There are few objective measures of gaming addiction, and their use may not be feasible on the scale of this study.

## Conclusion

Anxiety was a predictor of IGD, with statistically significant group differences in total anxiety, as well as the dimensions of physiological anxiety, social correlation, and sensitivity. The timely assessment of anxiety in children and adolescents, training social skills, and facilitating effective integration into society could be effective ways of reducing the incidence and impact of IGD. Future researches can explore sex-specific pathways to IGD, for example, if IGD prevalence in males and females could be predicted by different types of anxiety?

## Data Availability Statement

The original contributions presented in the study are included in the article/supplementary material, further inquiries can be directed to the corresponding author/s.

## Ethics Statement

The studies involving human participants were reviewed and approved by Biomedical Ethics Committee of West China Hospital of Sichuan University. Written informed consent to participate in this study was provided by the participants' legal guardian/next of kin.

## Author Contributions

XH and H-xS: conceptualization, methodology, writing—reviewing and editing, and writing original draft. H-qL: data collection, data analyses, and writing—reviewing and editing. W-jG: data collection, data analyses, and writing–reviewing. DL: methodology, data analyses, and writing—reviewing. J-jX: conceptualization, methodology, writing-reviewing, and supervision. All authors contributed to the article and approved the submitted version.

## Conflict of Interest

The authors declare that the research was conducted in the absence of any commercial or financial relationships that could be construed as a potential conflict of interest.

## Publisher's Note

All claims expressed in this article are solely those of the authors and do not necessarily represent those of their affiliated organizations, or those of the publisher, the editors and the reviewers. Any product that may be evaluated in this article, or claim that may be made by its manufacturer, is not guaranteed or endorsed by the publisher.

## References

[1] Kuss D. Internet gaming addiction: current perspectives. Psychol Res Behav Manag. (2013) 6:125–37. 10.2147/PRBM.S39476 24255603 PMC3832462

[2] Spekman M Konijn EA Roelofsma P Griffiths M. Gaming addiction, definition and measurement: a large-scale empirical study. Comput Human Behav. (2013) 29:2150–55. 10.1016/j.chb.2013.05.015

[3] Bass PF. Gaming addiction: when going online goes off-kilter. Contemp Pediatr. (2015) 32:16–23. 10.1089.2015.0034

[4] Poddar S Sayeed N Mitra S. Internet gaming disorder: application of motivational enhancement therapy principles in treatment. Ind J Psychiatry. (2015) 57:100–1. 10.4103/0019-5545.148540 25657471 PMC4314902

[5] Kuss DJ Griffiths MD Pontes HM. Chaos and confusion in DSM-5 diagnosis of internet gaming disorder: issues, concerns, and recommendations for clarity in the field. J Behav Addict. (2017) 6:103–09. 10.1556/2006.5.2016.062 27599673 PMC5520132

[6] Kishi T Tsunoka T Ikeda M Kawashima K Okochi T Kitajima T. Serotonin 1A receptor gene and major depressive disorder: an association study and meta-analysis. J Human Genet. (2009) 54:629–33. 10.1038/jhg.2009.84 19730445

[7] Darvesh N Radhakrishnan A Lachance CC Nincic V Tricco AC. Exploring the prevalence of gaming disorder and internet gaming disorder: a rapid scoping review. Syst Rev. (2020) 9:2–4. 10.21203/rs.2.19279/v2 32241295 PMC7119162

[8] Sarda E Bègue L Bry C Gentile D. Internet gaming disorder and well-being: a scale validation. Cyberpsychol Behav Soc Network. (2016) 36:102–07. 10.1089/cyber.2016.0286 27831752

[9] Young K. Understanding online gaming addiction and treatment issues for adolescents. Am J Family Ther. (2009) 37:355–72. 10.1080/01926180902942191

[10] Center CINI. Internet Statistical Reports: The 46th Survey Report (2018). Available online at: http://www.cnnic.com.cn/IDR/ReportDownloads/ (accessed December 25, 2012).

[11] Paulus FW Ohmann S Von Gontard A Popow C. Internet gaming disorder in children and adolescents: a systematic review. Devel Med Child Neurol. (2018) 11:132–140. 10.1111/dmcn.13754 29633243

[12] Association AP. Diagnostic and Statistical Manual of Mental Disorders (DSM-5). Washingtong, DC: American Psychiatric Association (2013).

[13] Wittchen HU Kessler RC Beesdo K Krause P Hofler MJH. Generalized anxiety and depression in primary care: prevalence, recognition, and management. J Clin Psychiatry. (2002) 63(Suppl. 8):24–34. 10.1093/her/17.1.85 12044105

[14] Hoge EA Ivkovic ALFG. Generalized anxiety disorder: diagnosis and treatment. BMJ. (2012) 345:e7500. 10.1136/bmj.e7500 23187094

[15] Katzman MA Bleau P Blier P Chokka P Kjernisted K Van Ameringen M. Canadian clinical practice guidelines for the management of anxiety, posttraumatic stress and obsessive-compulsive disorders. BMC Psychiatry. (2014) 14(Suppl. 1):S1. 10.1186/1471-244X-14-S1-S1 25081580 PMC4120194

[16] Kim NR Hwang SS Choi JS Kim DJ Demetrovics Z Király O. Characteristics and psychiatric symptoms of internet gaming disorder among adults using self-reported DSM-5 criteria. Psychiatry Invest. (2016) 13:58–66. 10.4306/pi.2016.13.1.58 26766947 PMC4701686

[17] Carras MC Shi J Hard G Saldanha IJ. Evaluating the quality of evidence for gaming disorder: a summary of systematic reviews of associations between gaming disorder and depression or anxiety %. plus one. (2020) 15:23–9. 10.1371/journal.pone.0240032 33104730 PMC7588081

[18] Dullur P Krishnan V Diaz A. A systematic review on the intersection of attention-deficit hyperactivity disorder and gaming disorder. J Psychiatric Res. (2021) 133:212–22. 33360866 10.1016/j.jpsychires.2020.12.026

[19] Wei HT Chen MH Huang PC Bai YM. The association between online gaming, social phobia, and depression: an internet survey. BMC Psychiatry. (2012) 12:92. 10.1186/1471-244X-12-92 22839747 PMC3545926

[20] Carlisle KL Neukrug E Pribesh S Krahwinkel J. Personality, motivation, and internet gaming disorder: conceptualizing the gamer. J Addict Offender Couns. (2019) 40:107–22. 10.1002/jaoc.1206925855820

[21] Király O Urbán R Griffiths MD Ágoston C Nagygyörgy K Kökönyei G. The mediating effect of gaming motivation between psychiatric symptoms and problematic online gaming: an online survey. J Med Int Res. (2015) 17:e88. 10.2196/jmir.3515 25855558 PMC4405620

[22] Marino C Canale N Vieno A Caselli G Scacchi L Spada MM. Social anxiety and internet gaming disorder: the role of motives and metacognitions. J Behav Addict. (2020) 9:617–28. 10.1556/2006.2020.00044 32750032 PMC8943663

[23] Smith JP Book SW. Anxiety and substance use disorders: a review. Psychiatric Times. (2008) 25:19–23. 10.1177/1039856215590053 20640182 PMC2904966

[24] Smith JP Book SW. Comorbidity of generalized anxiety disorder and alcohol use disorders among individuals seeking outpatient substance abuse treatment. Addict Behav. (2010) 35:42–5. 10.1016/j.addbeh.2009.07.002 19733441 PMC2763929

[25] Smith JP Randall CL. Anxiety and alcohol use disorders: comorbidity and treatment considerations. Alcohol Res. (2012) 34:414–31. 10.1159/000342488 23584108 PMC3860396

[26] Chen K Oliffe J Kelly M. Internet gaming disorder: an emergent health issue for men. Am J Mens Health. (2018) 12:1151–59. 10.1177/1557988318766950 29606034 PMC6131461

[27] Cao F Su L Liu T Gao X. The relationship between impulsivity and internet addiction in a sample of Chinese adolescents. Eur Psychiatry. (2007) 22:466–71. 10.1016/j.eurpsy.2007.05.004 17765486

[28] Podsakoff PM Mackenzie SB Lee JY Podsakoff NP. Common method biases in behavioral research: a critical review of the literature and recommended remedies. Appl Psychol. (2003) 88:879–903. 10.1037/0021-9010.88.5.879 14516251

[29] Siegel LJ Fletcher MG Anzem E. Diagnostic and statistical manual of mental disorders: encyclopedia of special education. Appl Psychol. (2003) 88:879–903. 10.1002/9780470373699.speced065525855820

[30] Pontes HM Griffiths M. Measuring DSM-5 internet gaming disorder: development and validation of a short psychometric scale. Comput Human Behav. (2015) 45:137–43. 10.1016/j.chb.2014.12.006

[31] Yam C Pakpour A Griffiths M Yau W Lo CM Ng J. Psychometric testing of three Chinese online-related addictive behavior instruments among Hong Kong University students. Psychiatric Quart. (2019) 90:117–28. 10.1007/s11126-018-9610-7 30328020

[32] Chen IH Ahorsu DK Pakpour AH Griffiths MD Lin CY Chen CY. Psychometric properties of three simplified Chinese online-related addictive behavior instruments among mainland Chinese primary school students. Front Psychiatry. (2020) 11:875–75. 10.3389/fpsyt.2020.00875 33101070 PMC7495180

[33] Ko CH Yen JY Chen SH Wang PW Chen CS Yen CF. Corrigendum to “Evaluation of the diagnostic criteria of internet gaming disorder in the DSM-5 among young adults in Taiwan”. J Psychiat Res. (2014) 57:185–85. 10.1016/j.jpsychires.2014.06.005 24581573

[34] Reynolds CR Richimond BO. What I think and feel: a revised measure of children's manifest anxiety. J Abnormal Child Psychol. (1978) 6:271–80. 10.1007/BF00919131 670592

[35] Castaneda A McCandless BR Palermo DS. The children's form of the manifest anxiety scale. Child Dev. (1956) 27:317–26. 10.2307/1126201 13356330

[36] Gigliotti E. Discovering statistics using spss. J Adv Nurs. (2007) 58:303–03. 10.1111/j.1365-2648.2007.04270_1.x

[37] Dreier M Wölfling K Duven E Giralt S Beutel M Müller K. Free-to-play: about addicted whales, at risk dolphins and healthy minnows. Monetarization design and internet gaming disorder. Addict Behav. (2017) 64:328–33. 10.1016/j.addbeh.2016.03.008 27178749

[38] Müller K Janikian M Dreier M Wölfling K Beutel M Tzavara C. Regular gaming behavior and internet gaming disorder in European adolescents: results from a cross-national representative survey of prevalence, predictors, and psychopathological correlates. Eur Child Adol Psychiatry. (2015) 24:565–74. 10.1007/s00787-014-0611-2 25189795

[39] Lopez-Fernandez O Ma HS Baguley T Griffiths M. Pathological video game playing in Spanish and British adolescents: towards the exploration of internet gaming disorder symptomatology. Comput Human Behav. (2014) 41:304–12. 10.1016/j.chb.2014.10.011

[40] Chang E Kim B. School and individual factors on game addiction: a multilevel analysis. Int J Psychol. (2020) 55:822–31. 10.1002/ijop.12645 31875984

[41] Karayagiz Muslu G Aygun O. An analysis of computer game addiction in primary school children and its affecting factors. J Addict Nurs. (2020) 31:30–8. 10.1097/JAN.0000000000000322 32132422

[42] Vollmer C Randler C Horzum MB Ayas T. Computer game addiction in adolescents and its relationship to chronotype and personality. Sage Open. (2014) 4:1–9. 10.1177/215824401351805434906014

[43] Kirby A Jones C Copello A. The impact of massively multiplayer online role playing games (MMORPGs) on psychological wellbeing and the role of play motivations and problematic use. Int J Mental Health Addict. (2014) 12:36–51. 10.1007/s11469-013-9467-9

[44] Lee C Kim O. Predictors of online game addiction among Korean adolescents. Addict Res Theory. (2017) 25:58–66. 10.1080/16066359.2016.1198474

[45] Esposito M Serra N Guillari A Simeone S Sarracino F Continisio G. An investigation into video game addiction in pre-adolescents and adolescents: a cross-sectional study. Medicina. (2020) 56:1–7. 10.3390/medicina56050221 32384823 PMC7279472

[46] Porter J Roy A Benson B Carlisi C Collins P Leibenluft E. Age-related changes in the intrinsic functional connectivity of the human ventral vs. dorsal striatum from childhood to middle age. Dev Cognit Neurosci. (2015) 11:83–95. 10.1016/j.dcn.2014.08.011 25257972 PMC6310902

[47] Ernst M Pine DS Hardin M. Triadic model of the neurobiology of motivated behavior in adolescence. Psychol Med. (2006) 36:299–312. 10.1017/S0033291705005891 16472412 PMC2733162

[48] Kardefelt-Winther D. A conceptual and methodological critique of internet addiction research: towards a model of compensatory internet use. Comput Human Behav. (2014) 31:351–54. 10.1016/j.chb.2013.10.059

[49] Giardina A Blasi MD Schimmenti A King DL Billieux J. Online gaming and prolonged self-isolation: evidence from italian gamers during the COVID-19 outbreak %. J Clin Neuropsychiatry. (2021) 18:65–74. 10.36131/cnfioritieditore210106 34909021 PMC8629072

[50] Dullur DP Krishnan V Diaz AM. A systematic review on the intersection of attention-deficit hyperactivity disorder and gaming disorder. J Psychiatric Res. (2020) 133:212–22. 10.1016/j.jpsychires.2020.12.026 33360866

[51] Ortiz D Griffiths M. Automatic mental processes, automatic actions and behaviours in game transfer phenomena: an empirical self-report study using online forum data. Int J Mental Health. (2014) 12:432–52. 10.1007/s11469-014-9476-3

[52] Ortiz de Gortari AB Griffiths MD. Prevalence and characteristics of game transfer phenomena: a descriptive survey study. Int J Human Comp Int. (2016) 32:470–80. 10.1080/10447318.2016.1164430

[53] Angelica B de Gortari O. Game transfer phenomena: origin, development, and contributions to the video game research field. Alison Attrill Smith. (2019) 13:532–56. 10.1093/oxfordhb/9780198812746.013.29

[54] Attrill-Smith A Fullwood C Keep M Kuss DJ. The Oxford Handbook of Cyberpsychology. Oxford: Oxford University Press (2019).

[55] Brand M Young K Laier C Wölfling K Potenza M. Integrating psychological and neurobiological considerations regarding the development and maintenance of specific internet-use disorders: an interaction of person-affect-cognition-execution (I-PACE) model. Neurosci Biobehav Rev. (2016) 71:252–66. 10.1016/j.neubiorev.2016.08.033 27590829

[56] Burleigh TL Griffiths MD Sumich A Stavropoulos V Kuss DJ. A systematic review of the co-occurrence of Gaming Disorder and other potentially addictive behaviors. Curr Addict Rep. (2019) 6:383–401. 10.1007/s40429-019-00279-7

[57] Laconi S Pires S Chabrol H. Internet gaming disorder, motives, game genres and psychopathology. Comput Human Behav. (2017) 75:652–59. 10.1016/j.chb.2017.06.012

[58] Gentile DA Choo H Liau A Sim T Li D Fung D. Pathological video game use among youths: a two-year longitudinal study. Pediatrics. (2011) 127:e319–29. 10.1542/peds.2010-1353 21242221

[59] Lemmens JS Valkenburg PM Peter J. Psychosocial causes and consequences of pathological gaming. Comp Human Behav. (2011) 27:144–52. 10.1016/j.chb.2010.07.01524001297

[60] Rooij A Schoenmakers TM Vermulst AA Regina JJM Eijnden VD Mheen D. Online video game addiction: identification of addicted adolescent gamers. Addict Res Theory. (2015) 106:205–12. 10.1111/j.1360-0443.2010.03104.x 20840209

