# Corrigendum: Differential effects of anxiety on internet gaming disorder: A large-scale cross-sectional survey

**DOI:** 10.3389/fpsyt.2022.969166

**Published:** 2022-07-18

**Authors:** Xia Huang, Hong-xia Shi, Hui-qin Li, Wan-jun Guo, Dan Luo, Jia-jun Xu

**Affiliations:** ^1^West China School of Nursing, West China Hospital, Sichuan University, Chengdu, China; ^2^Mental Health Center, West China Hospital, Sichuan University, Chengdu, China; ^3^Department of Pharmacy, West China Hospital, Sichuan University, Chengdu, China

**Keywords:** cross-sectional survey, internet gaming disorder, anxiety, addictive behavior, IgD

In the published article, there was an error in affiliation 1. Instead of “West China School of Nursing, Sichuan University, Chengdu, China,” it should be “West China School of Nursing, West China Hospital, Sichuan University, Chengdu, China.”

The authors apologize for this error and state that it does not change the scientific conclusions of the article in any way. The original article has been updated.

## Publisher's note

All claims expressed in this article are solely those of the authors and do not necessarily represent those of their affiliated organizations, or those of the publisher, the editors and the reviewers. Any product that may be evaluated in this article, or claim that may be made by its manufacturer, is not guaranteed or endorsed by the publisher.

